# 高级别胎儿型肺腺癌1例

**DOI:** 10.3779/j.issn.1009-3419.2019.03.12

**Published:** 2019-03-20

**Authors:** 川 黄, 超 马, 青峻 吴, 征 王, 耀光 孙, 鹏 焦, 文鑫 田, 瀚博 于, 宏峰 佟

**Affiliations:** 1 100730 北京，北京医院，国家老年医学中心，胸外科 Department of Thoracic Surgery, Beijing Hospital, National Center of Gerontology, Beijing 100730, China; 2 100730 北京，北京医院，国家老年医学中心，病理科 Department of Pathology, Beijing Hospital, National Center of Gerontology, Beijing 100730, China

**Keywords:** 肺肿瘤, 胎儿型腺癌, 预后, Lung neoplasms, Fetal adenocarcinoma, Prognosis

## Abstract

胎儿型肺腺癌属极其罕见的肺恶性肿瘤，2011年由国际肺癌研究协会、美国胸科学会和欧洲呼吸学会共同制定的国际多学科肺腺癌新分类将胎儿型肺腺癌归为浸润性腺癌的变异型，并将其分为低级别、高级别两类，二者临床病理特点和生物学行为不尽相同。本文报道高级别胎儿型肺腺癌1例，并结合文献总结胎儿型肺腺癌的临床病理特点。

## Abstract (en)

Fetal adenocarcinoma of the lung (FLAC) is an extremely rare subtype of lung cancer, accounting for only 0.1% to 0.5% of primary pulmonary malignancy. In 2011, international multidisciplinary classification of lung adenocarcinoma developed by the International Association for the Study of Lung Cancer (IALSC), the American Thoracic Society (ATS) and the European Respiratory Society (ERS) classified FLAC as a variant of invasive adenocarcinoma. FLAC has been further divided into low-grade fetal adenocarcinoma (L-FLAC) and high-grade fetal adenocarcinoma (H-FLAC) as these two categories exhibit different clinicopathological features and biological behaviors. Here we report a case of high-grade fetal adenocarcinoma and summarize clinicopathologic features of fetal lung adenocarcinoma.

## 1 病例资料

患者男性，55岁，2018年1月出现间断干咳，偶感左后背痛，无咳痰、咯血、发热、憋气等症状，饮食、大小便正常，体质量无明显下降，于2018年3月5日入院。患者长期在煤炭矿区工作，吸烟20年，10支/天-20支/天，戒烟3年，无饮酒，无家族性肿瘤、遗传病史。查体：左下肺野呼吸音明显降低，余肺野呼吸音正常。胸部计算机断层扫描（computed tomography, CT）（图 1）提示左肺下叶基底段可见巨大类圆形肿块影，大小约7 cm×8 cm，双侧肺门、纵隔未见明确肿大淋巴结。头颅核磁、骨扫描等全身检查未见远处转移。部分血清肿瘤标记物增高：癌胚抗原30.7 ng/mL（正常： < 5.0 ng/mL），血清骨胶素21-1 9.26 ng/mL（正常：0.1 ng/mL-3.3 ng/mL），神经原特异性烯醇化酶25.35 ng/mL（正常：0 ng/mL-16.3 ng/mL），余肿瘤标记物均在正常范围。入院诊断：左肺下叶肿物，肺癌可能性大。

**1:**
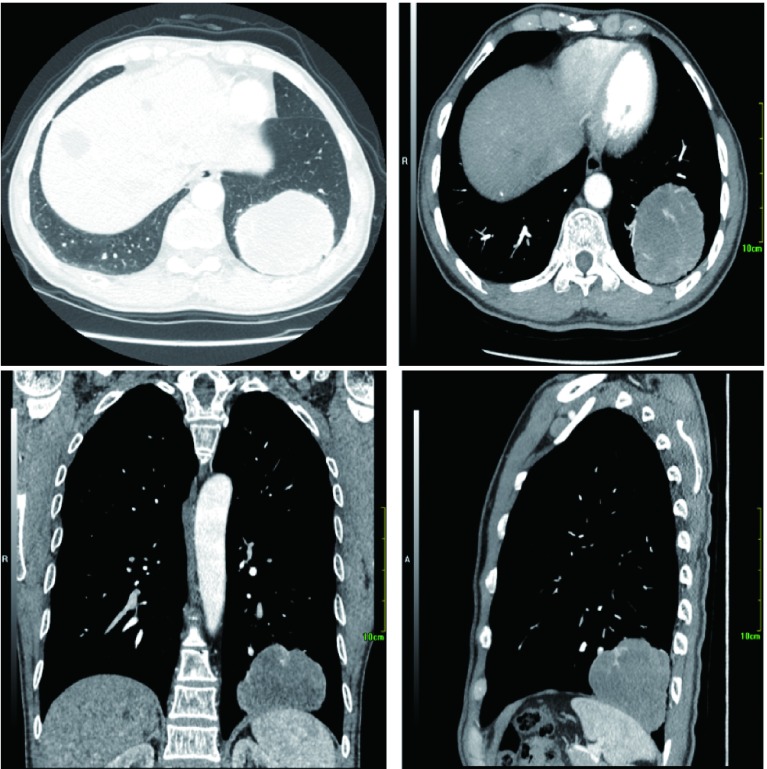
胸部CT显示左肺下叶基底段可见巨大类圆形肿块影，大小约7 cm×8 cm，密度欠均匀，增强扫描双期CT值分别为38 HU-58 HU、31 HU-55 HU，其内可见血管穿行，边缘呈浅分叶，境界清晰，相应支气管截断，局部胸膜肥厚。 Chest CT shows a heterogeneous well-defined peripheral mass in the basal segment of the left lower lobe. Size of the tumor is approximately 7 cm×8 cm and the dual-phase CT values of enhanced scan are 38 HU-58 HU and 31 HU-55 HU, respectively. The tumor is shallowly lobulated with blood vessels passing through, bronchial truncation and local pleural hypertrophy. CT: computed tomography.

2018年3月13日在全身麻醉下行胸腔镜左肺下叶切除、区域淋巴结清扫术，术中见肿物位于左肺下叶基底段，直径约8 cm，边界清楚，脏层胸膜表面可见脐凹征，切面呈灰黑色、质脆组织，内有大量坏死组织；肺门、隆突下、主-肺动脉窗及下肺韧带内可见多发肿大淋巴结。病理诊断：左肺下叶浸润性腺癌，中-低分化，形态符合高级别胎儿型腺癌（图 2），伴大片坏死，癌组织最大径8 cm，侵犯肺膜内弹力层，未见侵犯外弹力层（PL1），可见脉管癌栓，支气管及血管断端未见癌；第4组（0/1）、第5组（0/5）、第7组（0/7）、第8组（0/7）、第10组（0/8）、第11组（0/2）淋巴结均未见转移癌。*EGFR*基因18号-21号外显子29个位点均未检测出突变。免疫组化染色结果：β-catenin（+++，以胞膜阳性为主，图 3），CK7（+++），CK20（-），Ki67（热点区约70%-80%），P53（弥漫强表达，考虑错义突变型），TTF-1（+++），Napsin-A（-），c-met（2+），PD-1（示肿瘤间质淋巴细胞-），PDL-1（示肿瘤细胞-），Her2（2+），Ventana ALK（-），SYN（-），CgA（-），CD56（++），GPC-3（-），AFP（-），ER-β（++）。病理诊断：左肺下叶高级别胎儿型腺癌pT4N0M0，Ⅲa期。患者术后恢复良好，无并发症，术后行辅助化疗（方案：培美曲塞900 mg，d1+卡铂500 mg，d1）3个周期。规律随访至2018年10月，未见复发。

**2:**
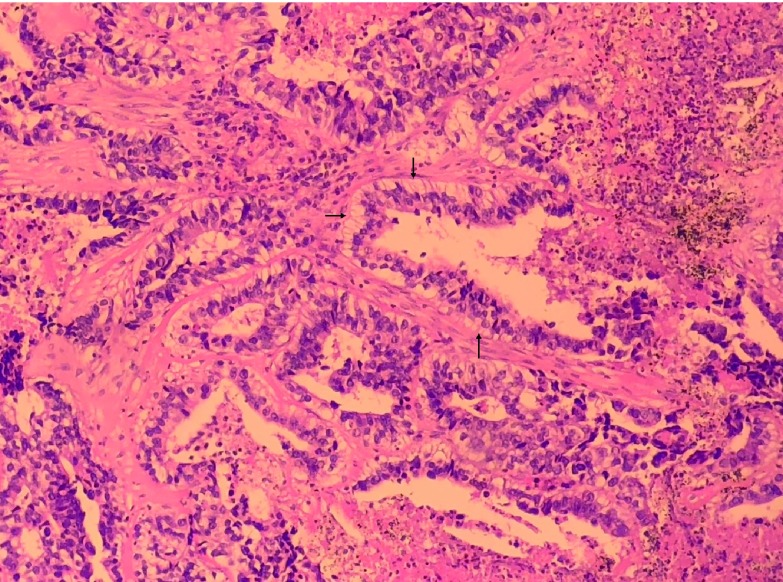
病理（HE染色，×400）：癌组织排列呈复杂腺腔样，癌细胞呈高柱状，胞浆富于糖原，细胞核异型明显，可见核下空泡（箭头处），未见桑葚体，伴多量坏死。 Haematoxylin and eosin staining, 400 times magnification: The tumor consists of complex glandular structures lined with glycogenrich high columnar cells, with obvious nuclear atypia and large amount of necrosis. Subnuclear vacuoles (arrow) are seen and no morule is found.

**3:**
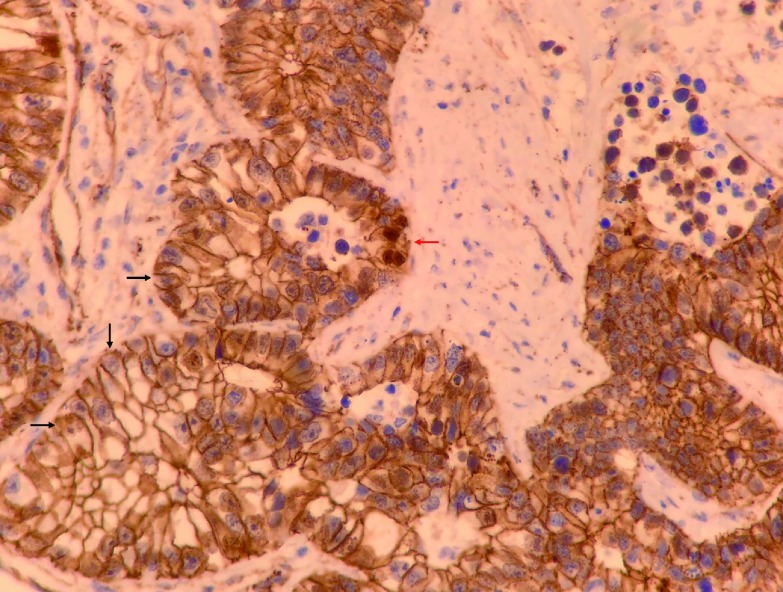
病理（免疫组化染色，×400）：*β*-catenin染色呈胞膜阳性（黑色箭头处），仅见极少量癌细胞呈胞核阳性（红色箭头处）。 Immunohistochemical staining of *β*-catenin, 400 times magnification: The tumor cells show membranous staining of *β*-catenin (black arrow). Very few tumor cells show nuclear staining of *β*-catenin (red arrow).

## 2 讨论

胎儿型肺腺癌（fetal adenocarcinoma of the lung, FLAC）属极其罕见的肺恶性肿瘤，仅占肺原发肿瘤的0.1%-0.5%，1982年由Kradin首次报道^[1]^，过去曾被归为“肺内胚层瘤”或“上皮型肺母细胞瘤”。2011年由国际肺癌研究协会（International Association for the Study of Lung Cancer, IALSC）、美国胸科学会（American Thoracic Society, ATS）和欧洲呼吸学会（European Respiratory Society, ERS）共同制定的国际多学科肺腺癌新分类^[2]^将FLAC归为浸润性腺癌的变异型，并将其分为低级别（low-grade fetal adenocarcinoma, L-FLAC）和高级别（high-grade fetal adenocarcinoma, H-FLAC）两类。FLAC病理特征为由富含糖原的无纤毛柱状细胞构成类似假腺样期的胎儿肺小管，细胞核具有轻微的不典型性，核底部含有较多空泡，腺管基底部或管腔内常可见由鳞状细胞样细胞形成的实性细胞球，即特征性桑葚小体（morule）。桑葚状细胞团常有神经内分泌分化，可表达NSE、CgA、Syn、CD56等标志物，电镜下可见部分肿瘤细胞质内有神经内分泌颗粒。FLAC可与腺癌、大细胞神经内分泌癌、小细胞癌、肠型腺癌、绒毛膜癌等其他病理类型混合发生，应根据肿瘤主要成分进行分类。

值得注意的是，L-FLAC和H-FLAC的临床病理特点和生物学行为有所不同。L-FLAC在FLAC病例中占大多数，常见于吸烟的青中年女性，病理镜下常有核下空泡、桑葚体等特征性改变，90%以上L-FLAC伴神经内分泌分化（表达Syn、CgA），研究^[3]^发现Wnt信号通路中的*β-catenin*基因的异常激活在L-FLAC的发病中发挥重要作用，免疫组化染色常可检测到细胞核和细胞质内β-catenin异常过表达。L-FLAC多表现为以局部生长为主的周围型肺部占位，直径1 cm-10 cm不等，边界清楚，肿瘤切面常呈灰白色、质脆，临床症状和胸部影像学无特殊性，确诊需病理诊断。L-FLAC属低度恶性肿瘤，很少出现远处转移，确诊时多为I期，首选手术切除，预后良好，5年总生存率可达80%^[4, 5]^。

H-FLAC多见于有重度吸烟史的中老年男性患者，表现为周围型肺部实性占位，病理特点为细胞核异型性更加明显，常缺少桑葚体，可转化成普通腺癌形态并出现坏死^[6]^。H-FLAC的病理诊断要求其主要成分为类似于假腺样期的胎儿肺，而当仅有部分胎儿型腺癌成分（不占主导地位）时，应诊断为其他类型腺癌含有胎儿型腺癌成分。H-FLAC的免疫组化染色特点为β-catenin多表达在胞膜而不在胞核，50%左右可伴神经内分泌分化，Syn、CgA、CD56阳性率为30%-60%，胚胎发育相关的AFP、GPC-3在H-FLAC（尤其是混合型H-FLAC）中的阳性率可高达90%^[7, 8]^。本例患者镜下癌组织排列呈复杂腺腔样，细胞核异型明显，可见核下空泡，未见特征性桑葚体，β-catenin染色呈胞膜阳性，符合H-FLAC病理特点。

H-FLAC恶性程度较L-FLAC高，确诊时常已出现区域淋巴结或远处转移，采用手术、化疗、放疗相结合的综合治疗，其远期疗效亦较好。Suzuki等^[7]^报道20例经手术切除证实的H-FLAC，其中病理分期为Ⅱ期者5例、Ⅲ期者6例、Ⅳ期2例，5年总生存率和无疾病生存率分别达82.4%和63.9%。本例患者虽未出现淋巴结转移，但肿瘤直径大、T分期较晚，病理分期为Ⅲa期，术后行辅助化疗，截至目前随访7个月，未见复发，需继续随访以观察远期疗效。
